# An accurate and efficient identification of children with psychosocial problems by means of computerized adaptive testing

**DOI:** 10.1186/1471-2288-11-111

**Published:** 2011-08-04

**Authors:** Antonius GC Vogels, Gert W Jacobusse, Symen A Reijneveld

**Affiliations:** 1 Cild Health, TNO, Leiden, The Netherlands; 2 Netherlands Forensic Institute, The Hague, The Netherlands; 3 Department of Health Sciences University Medical Center Groningen, University of Groningen, Groningen, The Netherlands

## Abstract

**Background:**

Questionnaires used by health services to identify children with psychosocial problems are often rather short. The psychometric properties of such short questionnaires are mostly less than needed for an accurate distinction between children with and without problems. We aimed to assess whether a short Computerized Adaptive Test (CAT) can overcome the weaknesses of short written questionnaires when identifying children with psychosocial problems.

**Method:**

We used a Dutch national data set obtained from parents of children invited for a routine health examination by Preventive Child Healthcare with 205 items on behavioral and emotional problems (n = 2,041, response 84%). In a random subsample we determined which items met the requirements of an Item Response Theory (IRT) model to a sufficient degree. Using those items, item parameters necessary for a CAT were calculated and a cut-off point was defined. In the remaining subsample we determined the validity and efficiency of a Computerized Adaptive Test using simulation techniques, with current treatment status and a clinical score on the Total Problem Scale (TPS) of the Child Behavior Checklist as criteria.

**Results:**

Out of 205 items available 190 sufficiently met the criteria of the underlying IRT model. For 90% of the children a score above or below cut-off point could be determined with 95% accuracy. The mean number of items needed to achieve this was 12. Sensitivity and specificity with the TPS as a criterion were 0.89 and 0.91, respectively.

**Conclusion:**

An IRT-based CAT is a very promising option for the identification of psychosocial problems in children, as it can lead to an efficient, yet high-quality identification. The results of our simulation study need to be replicated in a real-life administration of this CAT.

## Background

Many children suffer from behavioural and emotional problems [1-3] and these problems may seriously interfere with their daily functioning, now and later in life [4,5]. Yet many of these children remain untreated [5]. Early identification and treatment improves the prognosis of the children involved considerably [2,6].

Community-based preventive child healthcare (PCH) services, especially outreaching services, are in a unique position to identify such problems as early as possible. In the Netherlands, PCH professionals offer routine well-child care to the entire Dutch population to the age of about 14, free of charge. The early detection of children with psychosocial problems is an explicit part of their working package. In contrast to systems existing e.g. in the US, Dutch PCH does not offer treatment services. When (physical or psychosocial) problems are detected, children are referred to other parts of the healthcare system, especially to primary healthcare. Research has shown, however, that early identification in PCH is often far from perfect. For example, Brugman et al. showed that in Dutch PCH, about half of the children with a clinical CBCL Total Problem Score remained unnoticed when they were examined by a physician or nurse [1]. Other studies came to similar conclusions [7-11].

There are several possibilities to improve the identification of children with emotional and behavioural problems. Wiefferink et al. showed that using clear protocols and extensive staff training can lead to a significant increase in the number of children with problems identified and a decrease in the number of children incorrectly identified as having problems [12]. Other studies showed that using good questionnaires, to be filled in by parents, teachers or the children themselves, can also help to improve the quality of early identification [2,13-15]. However, in community-based PCH the time available for each individual child is limited. This means that questionnaires that are practicable in such settings, have to be easy to score and therefore short. Also, they must be easy for all parents to answer. Short questionnaires, unless they have a very narrow scope, tend to be less reliable and less valid than desirable [16]. Identification of problems based on such questionnaires is therefore error prone, resulting in too many false classifications.

Since the 1950s, new statistical models called Rasch or IRT (Item Response Theory) models have been developed which allow for Computerized Adaptive Testing (CAT), a short and efficient test procedure that does not compromise the accuracy of the test results. Originally, these models could only be applied to items with only two categories. This limited their application mainly to the field of intelligence testing and the assessment of school achievements [17]. In the last decades more widely applicable models have become available. This led to IRT-based test procedures in the field of quality of life measurements [18]. Some publications have been published describing the application of these models to the assessment of mental health problems [19-22].

Just like test procedures based on more traditional psychometric theories, IRT-based procedures help to determine the position of a person on some measurement scale, for instance on intelligence, school achievements or the level of psychosocial problems. In IRT that position is called the person location. IRT differs from traditional psychometrics in that it provides information about which items are relevant to use in an individual assessment and which are less useful. A simple example may illustrate this principle. Suppose in a particular arithmetic test, a child failed to give the correct answer to the question "How much is 2*3?" In that case it is probably not very useful to ask "How much is 34*17?" The latter question can help to distinguish between children on a higher position of the arithmetic ability scale, but will add little information for a child who failed to answer the first question correctly. Translating this to scales assessing emotional and behavioural problems, items indicating severe problems are not informative for children with no or few problems and items indicating less severe problems are not informative for children with severe problems.

With IRT it is possible to determine the severity of individual items; i.e. the position on the scale where it is informative. That position is called the item location [17]. This information can be used to shorten the test length in the following way. After each answer on a single question an estimation is made of the person's probable score, or person location. Then the available items are scanned in order to determine which item could improve the estimated person location. This continues until a previously defined accuracy has been reached. In practice this process is only possible with the aid of computers: Computerized Adaptive Testing (CAT) [23]. For CAT to be possible, the location of the items must be known in advance, before actual testing of an individual starts.

In this study we assessed whether CAT can also be used for a fast, short, yet high-quality identification of children with emotional and behavioural problems in community-based PCH. In order to do so, the following three questions will be answered:

1 Are the items of four questionnaires on emotional and behavioural problems suitable for an IRT-based CAT and, if so, which are the parameters (item locations) of the individual items, to be used in a CAT?

2 Which cut-off point results in a sensitive and specific distinction?

3 What are the validity and the specificity of such a CAT and how efficient is this procedure?

## Methods

### Data collection, population and measures

We used a data set collected in an earlier study [24] containing information about parent-reported problems of children aged seven to twelve. Data were collected in a two-step procedure. In the first step nine randomly selected regional PCH organizations were found willing to participate in our study. Second, parents who were invited for a regular care routine health examination of their child were asked to participate in the study and to fill in some questionnaires about emotional and behavioural disorders of their child. The study was approved by the Medical Committee of the Leiden University Medical Center.

Data from 2041 parents were available, that is 84% of all invited parents. Table 1 presents some demographic chracteristics of the respondents and non-respondents. The sample may be considered as representative for the population under care in Dutch PCH in this age group, with Cohen's W (a measure of effect size) varying from .002 for gender to .109 (for ethnic origin).

**Table 1:** Characteristics of the respondents and non-respondents

	Respondents	Non-respondents
	%	%

Child' gender (n (%))		

Male	1018 (50)	189 (49)

Female	1024 (50)	196 (51)

Child's age (mean (standard deviation))	9.7 (± 1.4)	9.7 (± 1.3)

Ethnic background (n (%))^#^		

Dutch	1694 (83)	216 (56)

non-Dutch	122 (6)	42 (11)

Unknown	204 (10)	127 (33)

Family composition (n (%))		

Two parents	1755 (86)	308 (80)

One parent	184 (9)	39 (10)

Other	102 (5)	39 (10)

Parental employment (n (%))		

No paid job	61 (3)	NA*

Two parents with paid job	1082 (53)	

One parent with paid job	1102 (54)	

Unknown	184 (9)	

Parental highest completed education (n (%))		

None or primary only (max. 8 yrs)	61 (3)	NA

Lower vocational (9 - 12 yrs.)	510 (25)	

Higher vocational (13 - 16 yrs)	633 (31)	

University/higher professional (17 yrs. and more)	714 (35)	

Unknown	122 (6)	

n	2041	385

* NA = not available

# P (Chi^2^) < .001 (for differences between respondents and non-respondents)

Each parent answered the Child Behavior Checklist (CBCL) and one out of three questionnaires: the Pediatric Symptom Checklist (PSC, n = 674), [25-27] the Strengths and Difficulties Questionnaire (SDQ, n = 707), [28-31] or a newly developed Dutch questionnaire on psychosocial problems for children in primary education, the PSYBOBA (n = 660) [32].

The SDQ was developed by Goodman as a screener for psychiatric problems in children, especially in community samples. Its validity and usability have been demonstrated in a large number of studies and in many countries, also in the Netherlands [24,33,34]. The SDQ contains 25 items and allows for the calculation of 5 subscales (Emotional Problems, Conduct problems, Problems with Peers, Hyperactivity and Prosocial Behavior. The first four subscales can be summed into a Total Problem scale. The PSC was developed by Jellinek and Murphy as a screener for psychosocial dysfunction. Its validity has also been well established. It allows for the calculation of a single Total Problem scale. At the time of the study no Dutch version was available. Therefore a Dutch version was developed inco-operation with the authors, based on three independent translations and back-translations [35]. This Dutch version was proven to be valid and reliable [24,36]. The PSYBOBA was developed in the Netherlands, as a screener for psychosocial problems among primary school children, specifically for Dutch PCH. Its 26 items also allow for the calculation of a single Total Problem scale. The validity of the PSYBOBA was shown to be perfectly comparable to that of the SDQ and PSC [24].

Which parent answered which of these three questionnaires was determined at random. The three sub-samples were similar with regard to the background characteristics mentioned above and with regard to the number of children being treated because of psychosocial problems and the number of children with a clinical score on the CBCL Total problem scale [24]. A clinical score was defined as a score above the 90^th ^percentile for specific age/gender groups in the Dutch normative sample, following the Dutch CBCL manual [37].

The PSC, SDQ and PSYBOBA were chosen for this study because there was evidence for their conceptual validity in relation to the kind of problems Dutch PCH aims to identify and because they met the requirements for use in the context of PCH: short, easy to administer and to score. Their validity in relation to a clinical CBCL Total problem scale score was shown to be similar, with sensitivity indices varying from 0.78 (PSC) to 0.86 (SDQ and PSYBOBA) and specificity indices from 0.90 to 0.91 [24]. The way in which we collected data led to an incomplete data matrix: the data for the PSC, the SDQ and the PSYBOBA are each available in about one third of the sample.

Finally, PCH professionals answered questions on current treatment status and emotional and behavioural problems of each child, based on medical records and on the routine health examination of the child, during which a small structured interview was done for the purpose of this study.

### Data analysis

We randomly divided the total sample in two subgroups. The first one, the calibration group (n = 1,650), was used to answer the first two questions (suitability of the items and determination of the cut-off point). The second, the validation group (n = 391), was used for the evaluation of the validity and efficiency. This evaluation in a separate group was done in order to prevent overestimation of validity and efficiency coefficients.

To assess the suitability of the items for an IRT-based CAT we assessed whether the items fitted the assumption of one-dimensionality. For this aim, we determined whether the items showed enough fit with the Partial Credit Model (PCM), one of the one-dimensional IRT models. Using this model for a CAT has the advantage that it results in scores on an interval measurement level [38]. We performed this assessment using the RUMM 2020 software (http://www.rummlab.com.au/), [39] as this can handle incomplete data matrices like ours. RUMM 2020 provides so-called outfit statistics for each item, that indicate to what extent each item fits the model. Items were considered suitable for CAT measurement if they had an outfit statistic smaller than 1.7.

Next, we calculated the item locations of the remaining items, using the same software. Additional file 1 presents an overview of the items, their means and standard deviations and -when not removed- item location. In order to determine whether the estimated item locations would be valid, independently from gender and ethnicity, we performed Differential Item Functioning (DIF) analyses for each item. We did this by multinomial logistic regressions, with the raw score on the item as the dependent variable. First, the estimated person location was the only predictor in the logistic regression model. Second, both gender, ethnicity and their interaction were added as predictors. Items were considered as showing DIF when these additional predictors had a significant effect and led to an increase of the explained variance of more than 3.5% [40].

Third, we determined an optimal cut-off point for the CAT scores, i.e. one which enables a good distinction between a non-clinical versus a clinical CBCL TPS. The CBCL TPS was used as the criterion measure, because it measures exactly the emotional and behavioural problems which Dutch PCH aims to identify and because both its concurrent and predictive validity have been widely established [41-44]. We simulated a CAT in the calibration group, using the answers on paper and pencil questionnaires as if they were given in a CAT and calculated the resulting person locations (CAT scores). We assume that in community-based PCH about 30 items is the maximum number feasible, and limited the number of items to be used in this CAT to 30. We used Fisher's information Index for the selection of the next item in the CAT [45]. A Bayesian approach with a right-skewed lognormal prior was used to estimate the person locations.

Using the scores from this simulation we did a Receiver Operating Characteristics analysis with a clinical CBCL TPS as criterion and chose that point that resulted in a specificity of 0.90 as cut-off point. The exact estimate of the person locations, however, will vary somewhat with the number of items used in the CAT. In order to assess the effect of this variation we repeated the analyses with a fixed number of 5, 10 and 20 items and also with no limit to the number of items, but continuing until the person locations had been estimated with 95% accuracy. In all these CATs the first item was chosen at random. We calculated the sensitivities and specificities for all these analyses and inspected the differences, in order to verify that the maximum of 30 items we used was a sensible one.

Finally, we evaluated the validity and efficiency of the CAT. The validity was assessed by means of a simulated CAT in the independent validation group. In this simulation we aimed to assess, with an accuracy of 95%, whether a person scored above or below the chosen cut-off point. In other words, the CAT was stopped when the 95% Confidence Interval of the estimated person location did no longer overlap with the chosen cut-off point. This procedure is known as clinical decision adaptive testing [46]. Again, the starting item was chosen at random, Fisher's Information index was used to select the next best item and a Bayesian approach was used to estimate the person locations. We assessed the validity of the estimated person locations by calculating the Area Under Curve (AUC), sensitivity and specificity with a clinical CBCL TPS and current treatment status as criteria. In order to enable some comparisons with results from other studies we also calculated kappas between the dichotomized CAT scores and dichotomized CBCL Total Problem Scale scores and being under treatment because of psychosocial problems. The efficiency of the procedure was evaluated by calculating the number of items needed in this simulated CAT and the number of respondents for whom the CAT resulted in 95% certainty on a score below or above the chosen cut-off point.

## Results

### Suitability of the items for an IRT-based CAT

Of the 205 non open-ended items in the four questionnaires 190 met the criteria for a CAT: they had an outfit of less than 1.7; 15 items were removed because of an outfit larger than 1.7 (Table 2). Most items that had to be removed came from the CBCL (13 out of 15).

**Table 2:** Items removed from the CAT because of an outfit > 1.7

Questionnaire	No	Item
CBCL	2	Allergies

CBCL	4	Asthma

CBCL	5	Behaves like a child of the opposite gender

CBCL	44	Nail-biting

CBCL	55	Overweight

CBCL	56d	Eye-problems

CBCL	56e	Skin problems

CBCL	56h	Other (psychosomatic) problems

CBCL	77	Sleeps more than peers

CBCL	97	Threatens other people

CBCL	98	Thumb-sucking

CBCL	107	Wetting him- or herself during the day

CBCL	108	Wetting his or her bed

SDQ	11	At least one good friend

SDQ	22	Steals from home, school or elsewhere

The Person Separation Index was 0.93, indicating a high reliability. The DIF analyses showed that almost all estimates were not modified by gender and ethnicity. Only 8 of the 190 items showed some DIF; 5 items from the CBCL, 2 from the PSC and 1 from both the SDQ and the PSYBOBA. Five of these items showed some DIF in relation to gender (sexual problems, running away, attacking others, being ill without physical cause and problems with teachers) and three in relation to ethnicity (tantrums, not being assertive, talking about suicide). Most of these problems have a very low prevalence; the percentages of parents reporting such problems being clearly or often present ranged from 0 to 5.8%. These items may therefore be expected to have a small overall impact on the final estimations. We therefore decided not to remove them.

**Figure 1:**
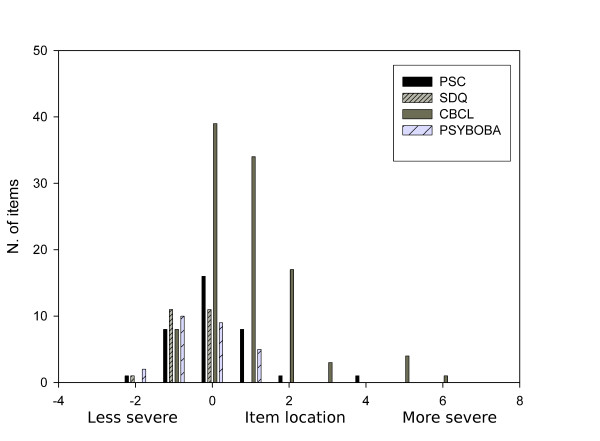
**Estimated locations (i.e. severity of items) of the items in the four questionnaires in the calibration sample**.

Figure 1 presents the estimated item locations calculated for the remaining items and split by questionnaire. As mentioned before, these item locations are indications of the level of severity. The most severe items on the right (concerning very serious problems) were items from the CBCL, which in general appeared to have more severe items than the other three questionnaires.

### Determining the cut-off point

**Figure 2:**
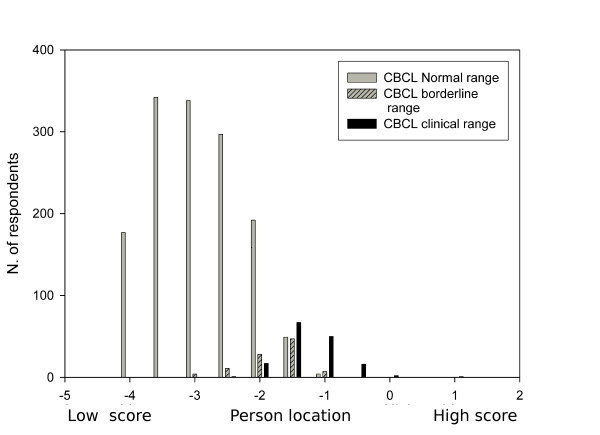
**Distribution of estimated person locations (i.e. low vs. high problem scores) in the calibration sample by CBCL classification Figure legend text**.

After the item locations had been estimated, we performed a CAT simulation on the calibration group with a fixed number of 30 items. Figure 2 presents the number of respondents by the calculated person location on the latent scale, by CBCL TPS, divided into normal, borderline or clinical. The ROC analysis showed that with a cut-off point of -1.9 the specified specificity of 0.90 was reached. The sensitivity for a clinical CBCL TPS at that point was 93%.

Table 3 presents the effects in terms of AUC, sensitivity and the specificity indices in relation to the use of different numbers of items in the CAT. The specificity shows little variation; using a fixed number of 5 or 10 items results in a decreased sensitivity. The results for a CAT with 20 or 30 items and for a CAT that continues until the 95% Confidence Interval no longer overlaps with the cut-off point are very similar.

**Table 3:** Sensitivity and specificity in relation to the number of items used to estimate persons' locations.

Criteria to stop the CAT	Sensitivity	Specificity	Area Under Curve
Maximum no of items: 30	93%	90%	0.97 (0.96 - 0.98)

Maximum no of items: 20	90%	89%	0.96 (0.95 - 0.97)

Maximum no of items: 10	79%	88%	0.92 (0.90 - 0.94)

Maximum no of items: 5	64%	88%	0.90 (0.84 - 0.89)

Estimation of person location with 95% accuracy	92%	90%	0.97 (0.96 - 0.98)

### Validity and efficiency

In the validation group, the ROC analyses showed that the CAT did very well in the identification of children with a clinical TPS; the AUC was 0.92 (CI: 0.85 - 0.99). With the chosen cut-off point sensitivity was 0.89 (CI: 0.71 - 0.97), with a specificity of 0.91 (CI: 0.87 - 0.93). Kappa was 0.53. Using treatment status as criterion the AUC was 0.74 (CI: 0.63 - 0.84). The sensitivity for current treatment status was 0.55 (CI: 0.37 - 0.72), with a specificity of 0.89 (CI: 0.85 - 0.92). Kappa was 0.32.

Overall, in relation to the CBCL TPS, the CAT selection procedure resulted in a correct classification of 91% of all children involved. The CAT resulted in a correct classification for the large majority of cases with normal (96%) or clinical scores (89%). However, 20 (77%) of the 26 cases with a score in the CBCL borderline range, had an elevated CAT score.

**Figure 3:**
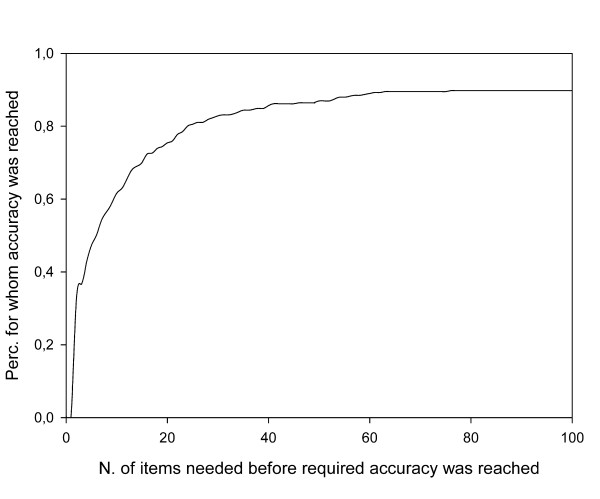
**Efficiency of the CAT procedure: percentage of persons for whom a score above or below the cut-off point could be estimated with 95% accuracy, by number of items used to achieve convergence**.

Figure 3 presents the number of items needed to reach convergence, i.e. to assess with 95% certainty whether the respondents had a true score below or above the chosen cut-off point of -1.9. In 40 cases (10%) convergence was not possible with less than 100 items. They had a mean person location of -1.88 (standard deviation, sd = 0.18); i.e. very near the chosen cut-off point. Their mean CBCL TPS was 28.4 (sd = 7.1).; 25% of them had a CBCL TPS in the borderline range; 5% in the clinical range.

For the other 351 cases, the mean number of items used was 11.5 (sd = 13.0). For 37% of the respondents the procedure converged with less than 5 items; for 57% up to 9 items were needed. For 74% up to 20 items were used and for 82% up to 30 items.

The mean CBCL TPS for respondents for whom less than 5 items were used in the CAT was 10.8 (sd = 10.5). We checked the convergence between the CBCL TPS based classification and the CAT classification for these respondents. In 98% of the cases the classification was identical. The CAT resulted in a score below cut-off point for 2 respondents with a clinical CBCL TPS and one respondent got a CAT score above the cut-off point with a CBCL in the normal range.

## Discussion

This study showed that IRT-based Computerized Adaptive Testing indeed resulted in an accurate, yet very efficient identification of children with psychosocial problems. Most of the items of the four questionnaires under investigation met the requirements of an IRT model, needed to incorporate them in a CAT. A simulation study showed that the procedure identified children with a clinical CBCL TPS with high sensitivity and specificity. For 90% of all cases we could determine with 95% certainty whether they had an elevated score. In order to achieve these results, on average only 11.5 items were needed. For more than half of the children less than ten items were needed.

There are, of course, other, more traditional techniques for reducing test length. However, in contrast to more traditional approaches, an IRT-based CAT provides high measurement quality, by adjusting items used in the assessment to the individual being tested. This has the additional advantage that this individual is not being confronted with items that are not relevant in his situation and that might be shocking for him or her. Therefore, the inclusion of items of the SDQ, PSC and PSYBOBA in the item pool used for this CAT offers the advantage that more items are available which are suitable for parents of children with no or few problems.

### Fit with the literature

Our finding that an IRT-based CAT can result in accurate assessments with far less items than tests based on traditional psychometrics is fully comparable to findings in other studies, applying IRT CAT techniques in the fields of intelligence and school achievement assessment, [16,17] and in the field of Quality of Life [18,22,47]. The first studies on the application of IRT models in the field of the identification of behavioural and emotional problems in paediatric care have now been published, [19-22] and these studies came to similar conclusions. Hill et al. [22] present a detailed analysis to assess the suitability of items from the Pediatric Quality of life Inventory for a CAT on distress but do not provide data on criterion validity. Compared to other validation studies regarding CAT and mental health, our study and the study by Gardner et al [20] are the only ones that focus on a rather broad concept, rather than on more specific problems, like Gardner [21] and Fliege et al. [19] Gardner et al. [20] used the PSC as criterion. As we used the more widely validated CBCL as one criterion, our study provides a stronger argument for the usefulness and validity of CAT-based procedures in the field of mental health.

Gardner et al [20] evaluated the extent to which a multidimensional adaptive test could be used to replicate screening decisions based on the Pediatric Symptom Checklist. He found a very high correspondence between the Adaptive PSC and the original 35 items PSC (kappa = 0.84), higher than the corresponding figure we found. The mean number of items he needed to achieve this was 12 items, out of 35, whereas we needed a mean of 12 items, to replicate the screening decision based on the 120 item CBCL. It is not exactly clear why he found a higher correspondence than we did. Our cut-off point was not chosen in order to maximize kappa, but had we done so, our kappa would still be lower than Gardner's. An explanation might be that Gardner limited himself to PSC items, whereas we used items from four questionnaires. Thus, in our study there is less overlap between the items in the CAT and the criterion measure. This is probably the main reason why Gardner's study resulted in a higher kappa.

### Strengths and Limitations

This study has several strengths but also limitations. A major strength is that it concerns a community-based sample of children with high response rates that is representative for the population under care. Furthermore, we used separate groups for the construction of the CAT and for its validation. A limitation of our study is that some of the items predicting the criterion, are part of the criterion itself, i.e. the CBCL items. In our view this does not hurt the validity of our conclusion regarding the quality of a short alternative for a longer questionnaire. Moreover, we simulated a CAT based on answers given to a full questionnaire, which is a deviation from the real practice set-up. A next stage will certainly be to evaluate the CAT in a set-up in which items are really presented using the CAT. Finally, although we had a rather large group, our validation group was relatively small, implying the need for a large-scale replication. Anyhow, our study provides a valid assessment of the potentials of an IRT-based CAT for PCH practice.

## Conclusion

The most important conclusion of our study is that IRT-based CAT appears to be a very feasible and promising tool to improve the identification of psychosocial problems in PCH. As such it earns a quick passing through to the daily practice of well-child care and maybe even of paediatric care in general, where there is a clear need for easy to use and sustainable high quality screening tools to increase the paediatrician's ability to identify children with mental health needs [48]. Before having a final pass to clinical practice, several aspects have to be studied more thoroughly, though. This in particular concerns the use of our simulated version in a real-life situation, with parents filling out the CAT on real computers. Currently, a beta-version for this aim is available on the internet, but this is Dutch only and protected by passwords and firewalls to preserve patient confidentiality. A formal assessment of this implementation in daily practice is the next step for research, which will focus among other things on acceptability and usability for parents and PCH professionals and privacy issues. Similarly, our findings have to be replicated in other settings and maybe using other item pools as well. Anyhow, this new technology may provide a push to improve the quality of the identification of psychosocial problems in PCH.

## Competing interests

The authors declare that they have no competing interests.

## Authors' contributions

AGCV and SAR were involved in all phases of the study: its design, data collection, analysis and reporting. GWJ contributed to the analysis of the data and reporting. All authors read and approved the final manuscript.

## Pre-publication history

The pre-publication history for this paper can be accessed here:

http://www.biomedcentral.com/1471-2288/11/111/prepub

## Supplementary Material

Additional file 1 **Items evaluated in the IRT analyses: content, mean, standard deviation and item location**. Data on items evaluated in the IRT-analyses, calibration sample. Click here for file
