# Supplementary material for: An accurate and efficient identification of children with psychosocial problems by means of computerized adaptive testing
Source: BMC Med Res Methodol. 2011 Aug 4;11:111. doi: 10.1186/1471-2288-11-111 (PMC3199909; doi:10.1186/1471-2288-11-111)
Supplement: Additional file 1 — Items evaluated in the IRT analyses: content, mean, standard deviation and item location. Data on items evaluated in the IRT-analyses, calibration sample. [file 1471-2288-11-111-S1.DOC]

### Additional file 1 – Items evaluated in the IRT analyses: content, mean^1^, standard deviation^1^ and item location^1^

| **Item^2^** | **content** | **mean** | **sd** | **item**  **location** | **outfit** |
| --- | --- | --- | --- | --- | --- |
| cbcl 1 | acts too young | 0.19 | 0.45 | -0.35 | 0.99 |
| cbcl 2 | allergy | 0.32 | 0.65 | removed | 2.71 |
| cbcl 3 | argues a lot | 0.56 | 0.60 | -1.37 | 0.84 |
| cbcl 4 | asthma | 0.14 | 0.45 | removed | 2.85 |
| cbcl 5 | behaves like a child of the opposite sex | 0.04 | 0.21 | removed | 1.74 |
| cbcl 6 | bowel movements outside toilet | 0.03 | 0.19 | 1.03 | 0.85 |
| cbcl 7 | bragging, boasting | 0.40 | 0.55 | -0.83 | 1.04 |
| cbcl 8 | can't concentrate | 0.50 | 0.65 | -1.44 | 0.86 |
| cbcl 9 | can't get mind off certain thoughts | 0.23 | 0.50 | -0.6 | 0.8 |
| cbcl 10 | can't sit still | 0.38 | 0.59 | -1.03 | 0.88 |
| cbcl 11 | clings to adults | 0.19 | 0.44 | -0.16 | 0.8 |
| cbcl 12 | complains of loneliness | 0.10 | 0.32 | 0.61 | 0.69 |
| cbcl 13 | confused, in a fog | 0.05 | 0.24 | 0.95 | 0.54 |
| cbcl 14 | cries a lot | 0.13 | 0.37 | 0.31 | 0.72 |
| cbcl 15 | Cruel to animals | 0.02 | 0.14 | 1.65 | 0.76 |
| cbcl 16 | cruelty, bullying to others | 0.06 | 0.25 | 1.13 | 0.63 |
| cbcl 17 | daydreams, gets lost in thoughts | 0.36 | 0.56 | -0.89 | 1.03 |
| cbcl 18 | deliberately harms self | 0.01 | 0.13 | 1.46 | 0.73 |
| cbcl 19 | demands a lot of attention | 0.41 | 0.61 | -1.14 | 0.73 |
| cbcl 20 | destroys own things | 0.05 | 0.24 | 0.76 | 0.5 |
| cbcl 21 | destroys other people's things | 0.04 | 0.20 | 1.54 | 0.55 |
| cbcl 22 | disobedient at home | 0.37 | 0.53 | -0.59 | 0.8 |
| cbcl 23 | disobedient at school | 0.16 | 0.38 | 0.49 | 0.78 |
| cbcl 24 | doesn't eat well | 0.26 | 0.51 | -0.72 | 1.24 |
| cbcl 25 | doesn't get along with other kids | 0.11 | 0.35 | 0.43 | 0.52 |
| cbcl 26 | doesn't seem to be guilty after misbehaving | 0.16 | 0.43 | -0.2 | 0.87 |
| cbcl 27 | easily jealous | 0.30 | 0.51 | -0.54 | 0.83 |
| cbcl 28 | eat or drinks things that are not for consumption | 0.01 | 0.12 | 1.73 | 0.59 |
| cbcl 29 | fears certain animals, situations …. | 0.23 | 0.51 | -0.71 | 1.31 |
| cbcl 30 | fears going to school | 0.03 | 0.17 | 1.74 | 0.69 |
| cbcl 31 | fears he/she might do something bad | 0.08 | 0.29 | 0.63 | 0.86 |
| cbcl 32 | Feels he/she has to be perfect | 0.44 | 0.61 | -1.24 | 1.23 |
| cbcl 33 | feels or complains that no one loves him/her | 0.14 | 0.39 | 0.19 | 0.59 |
| cbcl 34 | feels others are out to get him/her | 0.23 | 0.47 | -0.3 | 0.73 |
| cbcl 35 | feels worthless | 0.15 | 0.39 | 0.25 | 0.56 |
| cbcl 36 | get hurts a lot | 0.10 | 0.32 | 0.63 | 0.89 |
| cbcl 37 | gets in many fights | 0.06 | 0.25 | 1.2 | 0.65 |
| cbcl 38 | hangs around with others who get in trouble | 0.19 | 0.43 | -0.06 | 0.92 |
| cbcl 38 | gets teased a lot | 0.07 | 0.29 | 0.57 | 0.8 |
| cbcl 40 | hears sounds or voices that aren't there | 0.02 | 0.18 | 0.92 | 0.65 |
| cbcl 41 | impulsive, acts without thinking | 0.37 | 0.57 | -0.89 | 0.77 |
| cbcl 42 | would rather be alone than with others | 0.20 | 0.44 | -0.13 | 0.9 |
| cbcl 43 | lying or cheating | 0.15 | 0.38 | 0.4 | 0.81 |
| cbcl 44 | bites fingernails | 0.38 | 0.68 | removed | 2.34 |
| cbcl 45 | nervous, high-strung, or tense | 0.32 | 0.50 | -0.37 | 0.82 |
| cbcl 46 | nervous movements or twitching | 0.12 | 0.38 | -0.1 | 0.92 |
| cbcl 47 | nightmares | 0.15 | 0.38 | 0.17 | 1.06 |
| cbcl 48 | not liked by other kids | 0.13 | 0.36 | 0.43 | 0.65 |
| cbcl 49 | constipated, doesn't move bowels | 0.08 | 0.32 | 0.05 | 1.3 |
| cbcl 50 | too fearful or anxious | 0.12 | 0.36 | 0.14 | 0.8 |
| cbcl 51 | feels dizzy or lightheaded | 0.04 | 0.20 | 1.48 | 0.89 |
| cbcl 52 | feels too guilty | 0.09 | 0.32 | 0.38 | 0.74 |
| cbcl 53 | overeating | 0.13 | 0.39 | -0.22 | 1.13 |
| cbcl 54 | overtired without good reason | 0.11 | 0.33 | 0.66 | 0.73 |
| cbcl 55 | overweight | 0.14 | 0.42 | removed | 1.78 |
| cbcl 56,1 | phys. problems: pains - no headache | 0.13 | 0.38 | 0 | 0.97 |
| cbcl 56,1 | phys. problems: headache | 0.28 | 0.52 | -0.72 | 1.19 |
| cbcl 56,1 | phys. problems: nausea | 0.10 | 0.32 | 0.41 | 0.85 |
| cbcl 56,1 | phys. problems: with eyes | 0.10 | 0.38 | removed | 2.86 |
| cbcl 57,1 | phys. problems: skin problems | 0.16 | 0.44 | removed | 2.24 |
| cbcl 57,1 | phys. problems: stomach aches | 0.26 | 0.50 | -0.6 | 1.14 |
| cbcl 57,1 | phys. problems: vomiting | 0.06 | 0.26 | 0.78 | 0.89 |
| cbcl 57,1 | phys. problems: other | 0.11 | 0.41 | removed | 2.04 |
| cbcl 57 | physically attacks people | 0.06 | 0.24 | 1.33 | 0.62 |
| cbcl 58 | picks nose, skin, or other parts of body | 0.19 | 0.46 | -0.45 | 1.19 |
| cbcl 59 | plays with won sex parts in public | 0.01 | 0.14 | 1.04 | 0.67 |
| cbcl 60 | plays with own sex parts too much | 0.03 | 0.19 | 0.83 | 0.93 |
| cbcl 61 | poor schoolwork | 0.17 | 0.42 | -0.11 | 0.84 |
| cbcl 62 | poorly coordinated or clumsy | 0.16 | 0.41 | -0.03 | 0.84 |
| cbcl 63 | prefers being with older kids | 0.21 | 0.47 | -0.46 | 1.18 |
| cbcl 64 | prefers being with younger kids | 0.18 | 0.42 | -0.09 | 0.91 |
| cbcl 65 | refuses to talk | 0.10 | 0.33 | 0.37 | 0.77 |
| cbcl 66 | repeats certain acts over and over | 0.03 | 0.19 | 0.88 | 0.9 |
| cbcl 67 | runs away from home | 0.01 | 0.10 | 4.74 | 0.44 |
| cbcl 68 | screams a lot | 0.19 | 0.45 | -2.05 | 0.69 |
| cbcl 69 | secretive, keeps things to self | 0.26 | 0.52 | -0.62 | 0.86 |
| cbcl 70 | sees things that are not there | 0.02 | 0.16 | 1.22 | 0.8 |
| cbcl 71 | self-conscious or easily embarrassed | 0.24 | 0.47 | -0.35 | 0.83 |
| cbcl 72 | sets fires | 0.01 | 0.10 | 4.61 | 0.9 |
| cbcl 73 | sexual problems | 0.01 | 0.10 | 1.42 | 1.26 |
| cbcl 74 | showing off or clowning | 0.22 | 0.46 | -0.17 | 0.77 |
| cbcl 75 | too shy or timid | 0.32 | 0.51 | -0.52 | 1.15 |
| cbcl 76 | sleeps less than peers | 0.18 | 0.46 | -0.55 | 1.25 |
| cbcl 77 | sleeps more than peers during day and/or night | 0.04 | 0.22 | removed | 1.84 |
| cbcl 78 | plays with own faeces | 0.00 | 0.05 | 5.33 | 0.5 |
| cbcl 79 | speech problems | 0.06 | 0.26 | 0.56 | 1.46 |
| cbcl 80 | stares blankly | 0.04 | 0.21 | 1.33 | 0.53 |
| cbcl 81 | steals at home | 0.01 | 0.11 | 2.31 | 0.52 |
| cbcl 82 | steals outside the home | 0.01 | 0.10 | 2.25 | 0.58 |
| cbcl 83 | stores up too many things he/she does need | 0.11 | 0.39 | -0.26 | 1.3 |
| cbcl 84 | strange behaviour | 0.04 | 0.23 | 0.74 | 0.38 |
| cbcl 85 | strange ideas | 0.02 | 0.17 | 1.55 | 0.41 |
| cbcl 86 | stubborn, sullen or irritable | 0.43 | 0.56 | -0.86 | 0.77 |
| cbcl 87 | sudden changes in mood or feelings | 0.20 | 0.46 | -0.33 | 0.63 |
| cbcl 88 | sulks a lot | 0.40 | 0.54 | -0.66 | 0.9 |
| cbcl 89 | suspicious | 0.08 | 0.29 | 0.78 | 0.53 |
| cbcl 90 | swearing or obscene language | 0.15 | 0.37 | 0.64 | 0.84 |
| cbcl 91 | talks about killing self | 0.02 | 0.15 | 1.49 | 0.62 |
| cbcl 92 | talks or walks in sleep | 0.19 | 0.45 | -0.47 | 1.43 |
| cbcl 93 | talks too much | 0.35 | 0.57 | -0.94 | 0.94 |
| cbcl 94 | teases a lot | 0.13 | 0.36 | 0.46 | 0.74 |
| cbcl 95 | temper tantrums or hot temper | 0.25 | 0.51 | -0.62 | 0.69 |
| cbcl 96 | thinks about sex too much | 0.03 | 0.17 | 1.36 | 0.74 |
| cbcl 97 | threatens people | 0.01 | 0.10 | removed | 1.91 |
| cbcl 98 | thumb-sucking | 0.19 | 0.53 | removed | 3.72 |
| cbcl 99 | too concerned with being clean | 0.08 | 0.30 | 0.24 | 1.33 |
| cbcl 100 | troubles sleeping | 0.13 | 0.42 | -0.36 | 0.9 |
| cbcl 101 | truancy, skips school | 0.01 | 0.09 | 4.81 | 0.44 |
| cbcl 102 | underactive, slow moving or lacks energy | 0.08 | 0.31 | 0.27 | 0.83 |
| cbcl 103 | unhappy, sad, or depressed | 0.11 | 0.34 | 0.7 | 0.55 |
| cbcl 104 | unusually loud | 0.24 | 0.49 | -0.45 | 0.72 |
| cbcl 105 | uses alcohol or drugs | 0.00 | 0.08 | 2.03 | 0.66 |
| cbcl 106 | vandalism | 0.01 | 0.09 | 2.18 | 0.36 |
| cbcl 107 | wets self during the day | 0.02 | 0.15 | removed | 2.72 |
| cbcl 108 | wets the bed | 0.07 | 0.32 | removed | 2.68 |
| cbcl 109 | whining | 0.06 | 0.25 | 1.24 | 0.58 |
| cbcl 110 | wishes to be of opposite sex | 0.01 | 0.09 | 4.68 | 1.04 |
| cbcl 111 | withdrawn, doesn't get involved with others | 0.06 | 0.25 | 0.98 | 0.61 |
| cbcl 112 | worries | 0.20 | 0.43 | -0.03 | 0.73 |
| pbb 1 | excited, moves a lot | 0.71 | 0.75 | -2.05 | 1.19 |
| pbb 2 | easily distracted | 0.61 | 0.75 | -1.88 | 1.08 |
| pbb 3 | acts without thinking | 0.39 | 0.60 | -1.07 | 0.91 |
| pbb 4 | has friends | 0.13 | 0.37 | 0.17 | 1.03 |
| pbb 5 | is being bullied | 0.26 | 0.51 | -0.55 | 1.04 |
| pbb 6 | does not obey | 0.41 | 0.57 | -0.93 | 0.82 |
| pbb 7 | is assertive | 0.44 | 0.61 | -1.19 | 1.27 |
| pbb 8 | bullies other children | 0.17 | 0.40 | 0.26 | 0.97 |
| pbb 9 | has fits of anger | 0.29 | 0.57 | -0.9 | 0.9 |
| pbb 10 | little self confidence | 0.54 | 0.67 | -1.54 | 0.95 |
| pbb 11 | feels not liked by others | 0.34 | 0.58 | -0.95 | 0.83 |
| pbb 12 | considerate with other children | 0.25 | 0.52 | -0.63 | 1.24 |
| pbb 13 | withdrawn | 0.27 | 0.54 | -0.8 | 1.38 |
| pbb 14 | complains often about pains | 0.33 | 0.60 | -1.08 | 1.26 |
| pbb 15 | complies to rules | 0.49 | 0.62 | -1.3 | 1.08 |
| pbb 16 | is independent | 0.33 | 0.56 | -0.83 | 1.28 |
| pbb 17 | asks for attention in an annoying way | 0.31 | 0.57 | -0.85 | 0.69 |
| pbb 18 | behaves aggressively | 0.13 | 0.38 | 0.15 | 0.62 |
| pbb 19 | Problems with learning at school | 0.29 | 0.57 | -0.94 | 1.17 |
| pbb 20 | often not obedient | 0.21 | 0.45 | -0.1 | 0.8 |
| pbb 21 | sombre, unhappy | 0.13 | 0.38 | 0.16 | 0.51 |
| pbb 22 | anxious are afraid | 0.24 | 0.52 | -0.7 | 0.98 |
| pbb 23 | tells what bothers him/her | 0.42 | 0.59 | -1.06 | 1.33 |
| pbb 24 | cooperative | 0.17 | 0.40 | 0.32 | 1.16 |
| pbb 25 | satisfied with him or herself | 0.35 | 0.55 | -0.78 | 0.82 |
| pbb 26 | often quarrels with other children | 0.26 | 0.51 | -0.58 | 0.93 |
| psc 1 | complains about pains | 0.19 | 0.45 | -1.24 | 1.09 |
| psc 2 | spends more time alone | 0.32 | 0.65 | -1.32 | 1.2 |
| psc 3 | little energy | 0.56 | 0.60 | -0.25 | 1.13 |
| psc 4 | unable to sit still | 0.14 | 0.45 | -1.38 | 1.01 |
| psc 5 | trouble with teacher | 0.04 | 0.21 | 0.33 | 0.97 |
| psc 6 | less interest in school | 0.03 | 0.19 | -0.21 | 0.97 |
| psc 7 | acts as if driven by motor | 0.40 | 0.55 | -0.43 | 0.91 |
| psc 8 | daydreams | 0.50 | 0.65 | -0.72 | 1.11 |
| psc 9 | distracted easily | 0.23 | 0.50 | -2.18 | 0.93 |
| psc 10 | afraid of new situations | 0.38 | 0.59 | -1.38 | 1.09 |
| psc 11 | sad, unhappy | 0.19 | 0.44 | 0.02 | 0.78 |
| psc 12 | irritable, angry | 0.10 | 0.32 | -1.76 | 0.89 |
| psc 13 | Hopeless | 0.05 | 0.24 | 0.42 | 0.78 |
| psc 14 | trouble concentrating | 0.13 | 0.37 | -1.65 | 0.96 |
| psc 15 | less interested in friends | 0.02 | 0.14 | 0.08 | 1 |
| psc 16 | fights with other children, bullies | 0.06 | 0.25 | -0.03 | 0.8 |
| psc 17 | absent from school | 0.36 | 0.56 | 3.89 | 0.81 |
| psc 18 | school grades dropping | 0.01 | 0.13 | 0.24 | 0.81 |
| psc 19 | down on him or herself | 0.41 | 0.61 | -1.02 | 0.85 |
| psc 20 | visits doctor with doctor finding nothing wrong | 0.05 | 0.24 | 0.57 | 1.01 |
| psc 21 | trouble with sleeping | 0.04 | 0.20 | -0.18 | 1.11 |
| psc 22 | worries a lot | 0.37 | 0.53 | -0.65 | 0.87 |
| psc 23 | wants to be with you more than before | 0.16 | 0.38 | -0.31 | 0.85 |
| psc 24 | feels he or she is bad | 0.26 | 0.51 | 0.06 | 0.77 |
| psc 25 | takes unnecessary risks | 0.11 | 0.35 | 0.65 | 0.73 |
| psc 26 | gets hurt frequently | 0.16 | 0.43 | -0.24 | 1.21 |
| psc 27 | seems to be having less fun | 0.30 | 0.51 | 1.18 | 0.7 |
| psc 28 | acts younger than children of his or her age | 0.01 | 0.12 | 0.15 | 0.98 |
| psc 29 | does not listen to rules | 0.23 | 0.51 | -0.7 | 0.85 |
| psc 30 | does not show feelings | 0.03 | 0.17 | -1.03 | 1.13 |
| psc 31 | does not understand other people's feelings | 0.08 | 0.29 | -0.73 | 0.94 |
| psc 32 | teases others | 0.44 | 0.61 | -0.25 | 0.97 |
| psc 33 | blames others for his or her troubles | 0.14 | 0.39 | -0.84 | 0.84 |
| psc 34 | takes things not belonging to her or him | 0.23 | 0.47 | 0.38 | 0.9 |
| psc 35 | refuses to share | 0.15 | 0.39 | 0.37 | 0.73 |
| sdq 1 | considerate of other people's feelings | 0.10 | 0.32 | -0.34 | 0.9 |
| sdq 2 | Restless | 0.06 | 0.25 | -1.55 | 0.98 |
| sdq 3 | aches, sickness | 0.19 | 0.43 | -1.27 | 1.13 |
| sdq 4 | shares with other children | 0.07 | 0.29 | -0.72 | 1.13 |
| sdq 5 | temper tantrums | 0.02 | 0.18 | -0.87 | 0.82 |
| sdq 6 | Solitary | 0.37 | 0.57 | -1.29 | 1.37 |
| sdq 7 | Obedient | 0.20 | 0.44 | -1.11 | 0.93 |
| sdq 8 | many worries | 0.15 | 0.38 | -1.26 | 0.84 |
| sdq 9 | Helpful | 0.38 | 0.68 | -0.89 | 1.4 |
| sdq 10 | fidgeting, squirming | 0.32 | 0.50 | -1.62 | 1.12 |
| sdq 11 | at least one good friend | 0.12 | 0.38 | removed | 2.22 |
| sdq 12 | fights with other children, bullies | 0.15 | 0.38 | -0.09 | 0.94 |
| sdq 13 | unhappy | 0.13 | 0.36 | -0.5 | 0.75 |
| sdq 14 | liked by other children | 0.08 | 0.32 | -0.16 | 0.81 |
| sdq 15 | easily distracted | 0.12 | 0.36 | -2.12 | 1.15 |
| sdq 16 | Nervous, loses confidence | 0.04 | 0.20 | -1.65 | 1.07 |
| sdq 17 | kind to younger children | 0.09 | 0.32 | 0.1 | 1.51 |
| sdq 18 | lies or cheats | 0.13 | 0.39 | -0.17 | 0.79 |
| sdq 19 | bullied by other children | 0.11 | 0.33 | -0.53 | 0.98 |
| sdq 20 | volunteers to help others | 0.14 | 0.42 | -1.44 | 1.38 |
| sdq 21 | thinks before acting | 0.13 | 0.38 | -1.6 | 1.04 |
| sdq 22 | Steals | 0.28 | 0.52 | removed | 4.16 |
| sdq 23 | gets on better with adults | 0.10 | 0.32 | -0.51 | 1.12 |
| sdq 24 | many fears | 0.10 | 0.38 | -0.65 | 0.81 |
| sdq 25 | sees tasks through to the end | 0.16 | 0.44 | -1.78 | 1.08 |
| 1. means, stand deviation and item location as calculated on the calibration sample; means and standard deviations ona scale from 0 to 2 2. cbcl=Child Behavior Checklist; psc=Pediatric Symptom Checklist; pbb=PSYBOBA; sdq= Strengths & Difficulties Questionnaire | | | | | |
